# Identification of Candidate Genes Associated With Tolerance to Apple Replant Disease by Genome-Wide Transcriptome Analysis

**DOI:** 10.3389/fmicb.2022.888908

**Published:** 2022-05-09

**Authors:** Stefanie Reim, Traud Winkelmann, Alessandro Cestaro, Annmarie-Deetja Rohr, Henryk Flachowsky

**Affiliations:** ^1^Julius Kühn-Institut (JKI) - Federal Research Centre for Cultivated Plants, Institute for Breeding Research on Fruit Crops, Dresden, Germany; ^2^Woody Plant and Propagation Physiology Section, Institute of Horticultural Production Systems, Leibniz University Hannover, Hanover, Germany; ^3^Computational Biology Unit, Fondazione Edmund Mach, San Michele all’Adige, Italy

**Keywords:** RNA-seq validation, ‘M9’, *Malus* ×*robusta* 5, soil-borne disease, gibberellin biosynthesis, apple rootstocks, gene expression

## Abstract

Apple replant disease (ARD) is a worldwide economic risk in apple cultivation for fruit tree nurseries and fruit growers. Several studies on the reaction of apple plants to ARD are documented but less is known about the genetic mechanisms behind this symptomatology. RNA-seq analysis is a powerful tool for revealing candidate genes that are involved in the molecular responses to biotic stresses in plants. The aim of our work was to find differentially expressed genes in response to ARD in *Malus.* For this, we compared transcriptome data of the rootstock ‘M9’ (susceptible) and the wild apple genotype *M.* ×*robusta* 5 (Mr5, tolerant) after cultivation in ARD soil and disinfected ARD soil, respectively. When comparing apple plantlets grown in ARD soil to those grown in disinfected ARD soil, 1,206 differentially expressed genes (DEGs) were identified based on a log2 fold change, (LFC) ≥ 1 for up– and ≤ −1 for downregulation (*p* < 0.05). Subsequent validation revealed a highly significant positive correlation (*r* = 0.91; *p* < 0.0001) between RNA-seq and RT-qPCR results indicating a high reliability of the RNA-seq data. PageMan analysis showed that transcripts of genes involved in gibberellic acid (GA) biosynthesis were significantly enriched in the DEG dataset. Most of these GA biosynthesis genes were associated with functions in cell wall stabilization. Further genes were related to detoxification processes. Genes of both groups were expressed significantly higher in Mr5, suggesting that the lower susceptibility to ARD in Mr5 is not due to a single mechanism. These findings contribute to a better insight into ARD response in susceptible and tolerant apple genotypes. However, future research is needed to identify the defense mechanisms, which are most effective for the plant to overcome ARD.

## Introduction

Apple replant disease (ARD) is a worldwide economic risk for fruit tree nurseries and fruit growers. As a soil-borne disease complex, ARD occurs after replanting apple trees at a site previously occupied by apples. Characteristic symptoms of diseased plants are stunted shoot growth and root damage (Yim et al., 2017; Grunewaldt-Stöcker et al., 2019; Winkelmann et al., 2019). ARD leads to decreased and delayed fruit yields and reduced fruit and tree quality (Mazzola, 1998a; Mazzola and Manici, 2012).

Several microorganisms have been identified as causal agents and/or parts of this disease complex, such as *Nectriaceae* (Popp et al., 2019, 2020; Grunewaldt-Stocker et al., 2021; Manici et al., 2021), *Pythium* (Mazzola, 1998b)*, Rhizoctonia* (Mazzola, 1998b), *Streptomyces* (Mahnkopp-Dirks et al., 2021)*, Actinomycetes* (Popp et al., 2019; Radl et al., 2019) and *Fusarium* (Wang et al., 2018). It is assumed that the previous cultivation of apple in the same place leads to changes in the soil (micro)biome and impedes the soil’s capability to sustain the following apple culture (Winkelmann et al., 2019). However, the degree of ARD can vary on a regional, but also on a sub-plot scale due to differences in soil type and other abiotic and biotic factors (Simon et al., 2020).

Countermeasures such as crop rotation and soil disinfection are not practical due to concentration of producers in particular areas, but also due to environmental hazards or high costs (Winkelmann et al., 2019). Planting tolerant rootstocks would be a cost-effective and ecologically friendly strategy to overcome ARD, but such rootstocks are hardly available on the global market and rootstock breeding requires time and resources. Elucidating the genetic mechanisms underlying susceptibility/tolerance to ARD can greatly enhance the breeding efficiency. Establishment of molecular markers that are linked to ARD tolerance or which could be used as indicators for ARD severity in the plant would be useful for early selection of breeding progenies.

Recent studies revealed the induction of genes associated with biotic stress response in roots of plants grown in ARD-affected soil (Zhu et al., 2014; Weiß et al., 2017a; Reim et al., 2020; Rohr et al., 2020b). In particular, phytoalexins of the biphenyl biosynthetic pathway were demonstrably activated in response to ARD, which were shown to have an antifungal and antibacterial effect in plants (Chizzali et al., 2012). Individual phytoalexin compounds are differentially excreted by the plant, suggesting a controlled exudation process and thus an active influence of the plant on the soil microbiome and the severity of ARD (Busnena et al., 2021). Phenolic compounds were also shown to accumulate in response to ARD infected soil and may act as scavengers of reactive oxygen species (ROS; Leisso et al., 2018). These phenolic compounds may also lead to dark necrotic lesions of ARD infected roots, which are typical symptoms as described by Grunewaldt-Stöcker et al. (2019). Numerous flavonol metabolism genes as well as those involved in auxin, ethylene, jasmonate and cytokinin biosyntheses and signaling (Shin et al., 2014, 2016; Weiß et al., 2017a,b; Zhu et al., 2019; Reim et al., 2020) were also shown to be upregulated under ARD conditions. In particular, ethylene can induce the biosynthesis of phytoalexins derived from the phenylpropanoid pathway (Kamo et al., 2000; Chung et al., 2001; Ishigaki et al., 2004). Although there are already a number of studies on the response of the plant to an ARD infection at the level of gene expression, it is still unclear to what extent this response differs between susceptible and less susceptible or tolerant genotypes. Zhu and Saltzgiver (2020) gained first insights into the molecular response to inoculation and infection with *Pythium ultimum*, one member of the ARD pathogen complex, when comparing the transcriptomes of apple rootstock genotypes of different susceptibility levels. However, a comprehensive genome-wide transcriptomic comparison of apple genotypes that have faced the whole ARD complex was not carried out up to now.

In order to investigate the genetic response to ARD in more detail, whole transcriptome RNA sequencing was used in this study to generate transcriptome data from two apple genotypes with different susceptibility to ARD. The rootstock ‘M9’ was used as susceptible genotype, whereas the wild apple genotype *Malus ×robusta* 5 (Mr5) was used as ARD tolerant genotype. Potted plants of both genotypes were grown in ARD-affected soil and disinfected ARD soil, respectively. Roots were harvested and used to analyze the transcriptomic differences between the genotypes. Differentially expressed genes were identified and their putative function, and the involvement in biosynthetic pathways were predicted.

## Materials and Methods

### Plant Material

Plants of the apple rootstock ‘M9’ and the wild apple genotype *Malus ×robusta* 5 (Mr5) accession MAL0991 were propagated *in vitro via* axillary shoots on a modified MS medium (Murashige and Skoog, 1962; Rohr et al., 2020b). Mr5 is known to be tolerant, whereas ‘M9’ is susceptible to ARD (Reim et al., 2019). All *in vitro* cultures were grown at 24°C with 16 h of light provided by Philips MASTER TL-D 58 W/865 fluorescence tubes at a light intensity of 35–40 μmol m^−2^ s^−1^. Roots were induced after transferring five-week-old shoots to ½ MS medium supplemented with 2% sucrose and 4.92 μM IBA (Weiß et al., 2017a). For acclimatization to greenhouse conditions, all plants were transferred to peat substrate (Steckmedium, Klasmann-Deilmann GmbH, Geeste, Germany) and cultivated under covers to ensure high humidity. During acclimatization, plants were adapted to greenhouse conditions by gradually reducing the air humidity. After about 4 weeks, plants were transferred either to ARD-affected soil or disinfected ARD soil for the experiment and further cultivated in the greenhouse.

### Soil Material and Experimental Setup

ARD-affected soil from the experimental orchard of JKI Dresden-Pillnitz (51°00′01.6″N 13°53′14.7″E, Germany) was used. The soil is a luvisol with loamy sand to sandy loam soil texture. In this orchard, apple had been grown for several decades. ARD infection of the soil has already been proven in another trial (Reim et al., 2019). After old apple trees had been uprooted, soil was taken directly from tree holes at a depth of 5–35 cm. The soil was sieved through an 8 mm mesh and half of the volume was γ-irradiated with a minimum dose of 10 kGy (recorded dosages: minimum 10.87 kGy, maximum 31.96 kGy, BGS Beta-Gamma-Service, Wiehl, Germany) by which most fungi, bacteria and invertebrates are killed (McNamara et al., 2003). Hereafter, the non-irradiated ARD-affected soil will be denoted as ARD soil and the γ-irradiated, disinfected ARD soil as γARD soil.

Five representative plants per genotype were planted into plastic pots (one per 0.4 l pot) containing ARD soil, whereas five additional plants per genotype were planted into γARD soil. The soil was supplemented with 2 g L^−1^ of the slow-release fertilizer Osmocote® Exact® 3–4 M (16 + 9 + 12 + 2 MgO, Everris International B.V., Geldermalsen, Netherlands). All 20 plants were randomly arranged on a greenhouse table and cultivated at 18°C–20°C (day) and 16°C–18°C (night), 70% air humidity with a minimum of 14 h of daylight. Plant protection was carried out according to horticultural practice.

### Sample Preparation and RNA Extraction

Eight weeks after the plants had been planted in ARD soil or γARD soil, fine root tissue was harvested and immediately frozen in liquid nitrogen. Total RNA was extracted from 100 mg of frozen root tissue using the InviTrap Spin Plant RNA Mini Kit (Stratec, Birkenfeld, Germany) according to the manufacturer’s instructions. Genomic DNA was removed with DNase I (Thermo Scientific, Waltham, MA, United States) following the manufacturer’s instructions. RNA concentration and quality of RNA were determined spectrophotometrically (NanoDrop 2000c, Peqlab, Erlangen, Germany) and on an 1% agarose gel. The quality of the RNA was determined using the Agilent 2,100 Bioanalyzer and revealed a RNA Integrity Number (RIN) of all samples of >7.

### Strand-Specific RNA-Seq Library Preparation and RNA Sequencing

Three biological replicates per genotype (‘M9’ and Mr5) and soil variant (ARD soil and γARD soil) were selected for RNA sequencing. Construction of the RNA-seq libraries as well as RNA sequencing were performed by Eurofins Genomics (Ebersberg, Germany) and ATLAS biolabs (Berlin, Germany), respectively. Sequencing was performed on an Illumina HiSeq 2,500 instrument to generate 100 bp paired-end reads with 23 to 26 million read-pairs per sample. Quality control and read statistics for these 12 samples were determined by FastQC. Low quality reads were discarded.

### RNA-Seq Data Analysis

Reads passing the default Illumina quality filter procedure (chastity filter) were mapped to the reference transcriptome of *Malus* ×*domestica* cv. ‘Golden Delicious’ v.1.1 (Daccord et al., 2017). Raw data were checked for quality with trimmomatic software (version 0.39)^1^ using following trimming steps: SLIDINGWINDOW 20:30; ILLUMINACLIP; MINLEN 50 (Bolger et al., 2014). Reads were mapped by means bowtie2 aligner vers. 2.3.4.1 (Langmead and Salzberg, 2012) and read counts were obtained using htseq software vers. 0.9.1 (Anders et al., 2014). Differential gene expression was obtained by using the DESeq R package vers. 3.0.2 (Love et al., 2014) comparing ARD soil to γARD soil condition for each genotype, separately. Values of *p* for the statistical significance of the log_2_ fold change (LFC) were adjusted for multiple testing with the Benjamini–Hochberg correction for controlling the false discovery rate of <10% (Benjamini and Hochberg, 1995). Since the quality checks gave warnings about the amount of PCR duplicates, an additional analysis by removing these hypothetical PCR duplicates (rmdup) was done.

### Gene Enrichment and Functional Analysis

Assignment and visualization of the differentially expressed genes (DEGs) to pathways were carried out with MapMan (Thimm et al., 2004) using the *Malus domestica* mapping file (Mdomestica_196.txt).^2^ Significantly overrepresented gene categories for Mr5 and ‘M9’ were identified using Wilcoxon test and visualized in PageMan using MapMan 3.5.0 and mappings for *Malus ×domestica*. This feature tests whether the median fold-change within the respective ontological group was the same as the median fold-change of the complete collection of genes under analysis (Usadel et al., 2006).

The function of each single gene was determined based on the Gene IDs using the NCBI ‘Batch Entrez’ online tool^3^ to download the corresponding records of the *Malus ×domestica* UniGene database.

Pathway analysis of the genes was performed using the KEGG (Kyoto encyclopedia of genes and genomes) webtool.^4^ The input data are provided in Supplementary Table 1. The protein–protein interaction (PPI) networks for the DEGs were detected based on using the STRING database.^5^ Given a list of the DEGs as input (Supplementary Table 2) and the respective difference of fold change values between ‘M9’ and Mr5, STRING searched the proteins with direct interactions and generated the PPI network. The STRING output was visualized using Cytoscape (ver 3.8; Shannon et al., 2003).

### RT-qPCR Validation

To validate the RNA-seq results, 30 DEGs were selected that showed remarkable differences in gene expression between ‘M9’ and Mr5 and were assumed to play a possible role in the defense response to ARD. In addition, the three genes *Biphenyl synthase 3* (*BIS3*), *biphenyl 4-hydroxylase* (*B4H*), and *ethylene-responsive transcription factor 1B-like* (*ERF1B*) that had been shown in previous studies to be suitable biomarkers for early ARD diagnosis were analyzed (Reim et al., 2020; Rohr et al., 2020a). For the selected 30 candidate genes, primers were generated using Primer3 tool with the following parameters: primer length 18–24 bp, amplification product 100–200 bp, T_M_ = 59°C–61°C, CG content 40%–60% (Untergasser et al., 2012). A full list of all primers is provided in Supplementary Table 3. Primer pair specificity was firstly validated *in silico* using the software program FastPCR v6.6 (PrimerDigital Ltd., Helsinki, Finland; Kalendar et al., 2017) by calculating theoretical PCR results using the *Malus* ×*domestica.*v1.0.consensus_CDS database obtained from http://www.rosaceae.org. Primer sequences with proven specificity to the target gene sequence were further validated on an iCycler iQ Real Time PCR Detection System (Bio-Rad). RT-qPCR was performed using the Maxima SYBR Green master mix (Fisher Scientific, Schwerte, Germany) and final primer concentrations of 75 nM with an initial denaturation of 3 min at 94°C followed by 40 cycles of 1 min at 94°C, 1 min at 60°C, and 1 min at 72°C. The specificity of PCR products was analyzed by melt-curve analysis of 55°C to 80°C with a increment of 0.5°C for 10 s each step. Positively tested primers were subsequently validated for gene expression. Each sample was pooled containing two biological replicates and analyzed with three technical replicates by RT-qPCR as described above. For the analysis of the biomarker genes *BIS3*, *B4H*, and *ERF1B*, each sample was analyzed with five biological replicates and two technical replicates. The elongation factor 1-α [MDP0000304140], elongation factor 1β-like [MDP0000903484], tubulin beta chain [MDP00009551799], ubiquitin-conjugating enzyme E2 10-like [MDP0000140755] and actin-7 [MDP0000774288] were used as reference genes according to Flachowsky et al. (2010), Perini et al. (2014), Weiß et al. (2017a,b). The reference genes were validated according to their stability using NormFinder (Andersen et al., 2004). A dilution series (1,10, 1:100, 1:1000, and 1:2000) was created from equal amounts of cDNA of each sample. The calibration curve was used to calculate the amplification efficiencies within the CFX manager software according to Pfaffl (2001). The relative gene expression levels were calculated according to Pfaffl (2001) using also the CFX manager software. Pearsons’ correlation between the RNA sequencing data and the RT-qPCR data was calculated using SAS ver. 8 (SAS, Cary, United States).

## Results

### Growth Data Support the Divergent ARD Sensitivity of Both Genotypes

**Figure 1:**
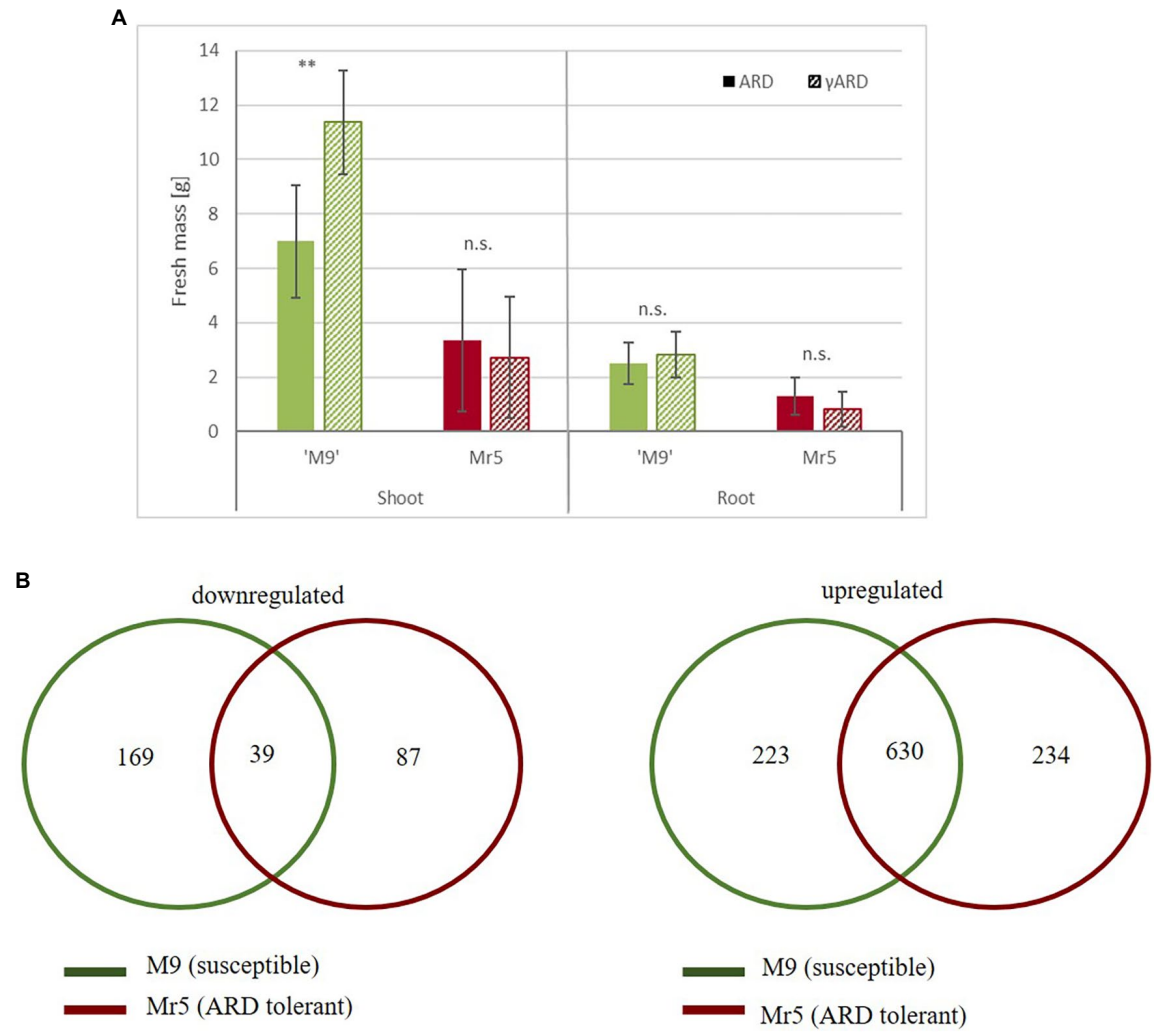
Biomass and gene expression comparisons between the susceptible rootstock ‘M9’ and the ARD tolerant wild apple genotype Mr5 in response to ARD. **(A)** Fresh mass (*g*) of ‘M9’ (green) and Mr5 (red) after cultivation in ARD soil and γARD soil (*n* = 5; given are means and SD). Comparisons between ARD and γARD variants were analyzed by ANOVA (n.s. = not significant; ** = *p* < 0.01). **(B)** The Venn diagrams indicate the number of common and exclusive DEGs (threshold = 1), separately for the downregulated and upregulated genes. DEGs exclusively detected in ‘M9’ are indicated in the green circle, DEGs exclusively detected in Mr5 are indicated in the red circle. Some DEGs were counted twice, because they were upregulated in one genotype and downregulated in the other. Thus, the overall sum of DEGs of both Venn diagrams is higher than the total DEG number.

After 8 weeks of cultivation in ARD soil or gamma-ARD soil in the greenhouse, shoot and root biomass of ‘M9’ and Mr5 were determined. The susceptible rootstock ‘M9’ showed significantly lower shoot mass in ARD soil (7 g) than in the disinfected γARD soil (11 g) (Figure 1A). The reduction in root biomass was less pronounced: only 12% lower root biomass was recorded after cultivation in ARD soil compared to γARD soil. In contrast, little difference was observed between ARD and γARD soil for the ARD tolerant wild species Mr5, which grew much slower, overall.

### Reads Processing and Identification of Differential Expressed Genes

RNA-seq of the 12 root samples resulted in 549.6 million raw reads, with an average of 46 million 100-bp single ended reads per sample. After quality control, 494.1 million reads were mapped to the apple reference genome (*Malus* ×*domestica* cv. ‘Golden Delicious’ v1.1) and assigned to 72,569 gene IDs (Reim et al., 2022, Dataset 1). In the susceptible rootstock ‘M9’ 2,697 genes were upregulated (log_2_ fold change, LFC > 1), whereas 1,411 genes were downregulated (LFC < −1). In the ARD tolerant wild apple genotype Mr5 2,985 genes were upregulated, but 1,836 genes were downregulated. Taking into account a mean expression level for ‘M9’ and *M.* ×*robusta* 5, using a cut off value of >1.0 and < −1.0 [log_2_ fold change, LFC] and a significance level of *p* < 0.05, 1,206 differentially expressed genes (DEGs) were observed. 169 DEGs were downregulated in ‘M9’ only and 87 DEGs were downregulated in Mr5 only (Figure 1B). However, most of the DEGs were upregulated (234 DEGs in Mr5 only and 223 DEGs in ‘M9’ only). For detailed information see Reim et al. (2022), Dataset 2.

### Functional Categorization of the DEGs

Using MapMan, the 1,206 DEGs were assigned to 31 functional categories (BINs). In the case of 19 DEGs, the assignment was not clear because they were assigned twice to two different groups. For detailed information about the functional groups (BINs), their similarity to other genes or proteins of *Arabidopsis thaliana* or other plants see Jeandet et al. (2014), Dataset 2. For 304 DEGs, no functional assignment was possible. 146 DEGs were assigned to ‘miscellaneous enzyme families (MISC)’, 130 DEGs to ‘RNA’ and 97 DEGs to ‘protein’. The functional groups ‘transport’ (81), ‘signaling’ (78), ‘hormone (74) and secondary metabolism (60)’ and ‘stress’ (59) contained 59 to 81 genes. The remaining 196 DEGs were associated to diverse functional groups.

**Figure 2:**
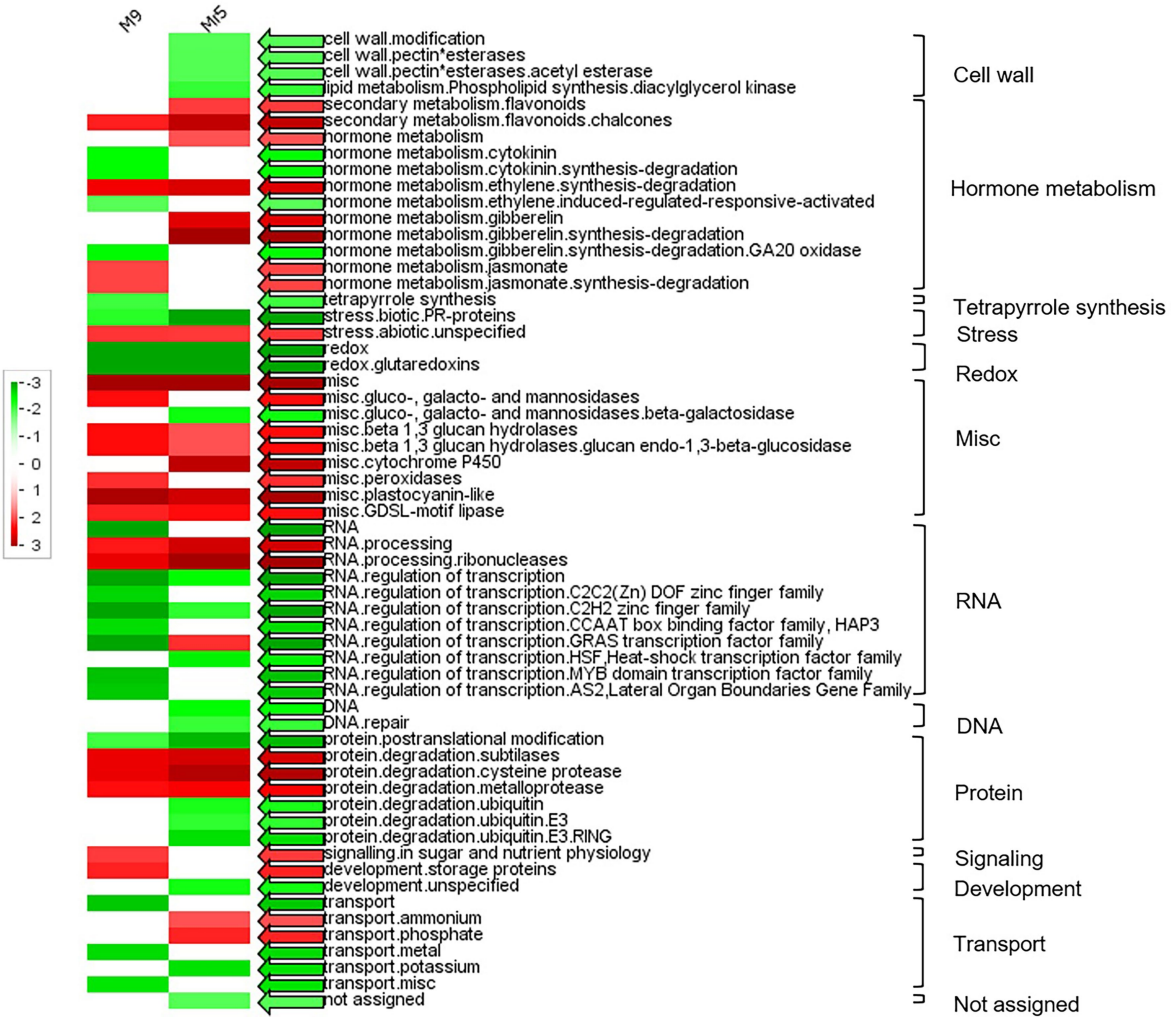
PageMan display of significantly represented functional categories that were differentially regulated in roots of the susceptible rootstock ‘M9’ and the ARD tolerant wild apple species *M.* ×*robusta* 5 (Mr5) plants after cultivation in ARD soil and compared to γARD soil. Colored boxes indicate statistically significant groups (*p* < 0.05) after Wilcoxon test. The results are displayed as red-colored BINs (significantly over represented), green-colored BINs (significantly under represented) and white-colored BINs (not significant). The colored arrows indicate the highest significance found for the respective functional category. The MapMan annotations of the gene classes were displayed alongside each row and the main functional categories are displayed in the right. Non-significant categories are not displayed.

A PageMan analysis was carried out to obtain a statistics-based overview of pathways altered in ‘M9’ and Mr5 in response to ARD. The results are illustrated in Figure 2. As emphasized before, the number of upregulated DEGs was higher than that of the downregulated DEGs. Significantly overrepresented upregulated genes (Figure 2, red arrows) were found in 23 functional groups (Figure 2). The most noteworthy upregulated gene groups in ‘M9’ were ‘hormone metabolism.jasmonate’, ‘misc.peroxidase’ and ‘misc.ß-1,3 glucan hydrolases’. The most noteworthy upregulated gene groups in Mr5 were ‘hormone metabolism.gibberellin’ and ‘secondary metabolism.flavonoids’. In 34 functional groups, downregulated DEGs were overrepresented (Figure 2, green arrows). Outstandingly downregulated genes in Mr5 belonged to the groups ‘cell wall’, ‘stress.biotic.PR-proteins’ and ‘protein.degradation.ubiquitin’. In ‘M9’, the most noteworthy downregulated functional group was ‘RNA.regulation of transcription’.

### Pathway Enrichment Analysis of DEGs

**Figure 3:**
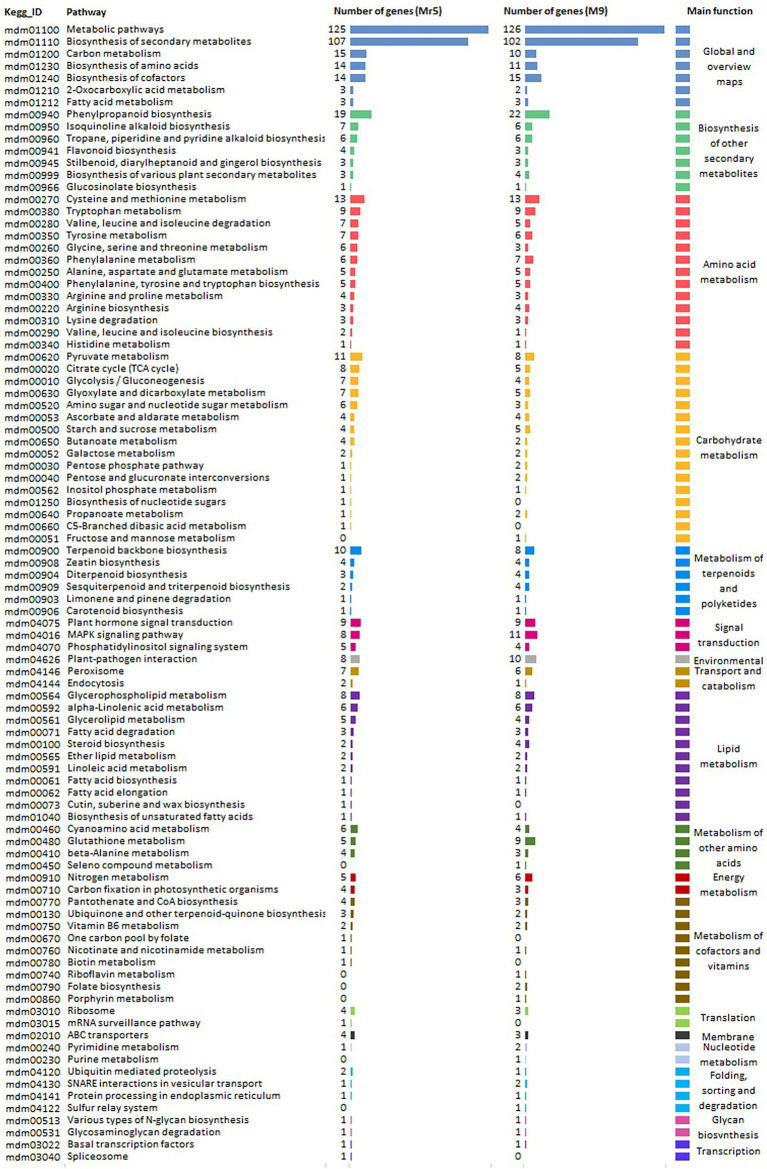
Kyoto Encyclopedia of Genes and Genomes (KEGG) pathway enrichment analysis for 1,206 DEGs identified based on a log_2_FC > 1 with a significance level of *p* < 0.05. The DEGSs were identified for the ARD tolerant wild apple cultivar Mr5 and the susceptible rootstock ‘M9’ in root material after cultivation in ARD soil and γARD soil. 600 DEGs of Mr5 and 575 DEGs of ‘M9’ were assigned to significantly enriched pathways of 18 different major functions which are represented by a various color.

600 DEGs from Mr5 and 575 DEGs from ‘M9’ were assigned to different KEGG pathways (Figure 3). For the ARD tolerant genotype Mr5 125 DEGs belonged to the ‘metabolic pathway’ and 107 DEGs to ‘biosynthesis of secondary metabolites’. Further 19 DEGs from Mr5 were assigned to ‘phenylpropanoid biosynthesis’, and 15 DEGs to ‘carbon metabolism’. 14 DEGs from Mr5 belong to ‘biosynthesis of amino acids’, and 14 to ‘biosynthesis of cofactors’. Further 13 DEGs of Mr5 were assigned to ‘cystein and methionine metabolism’, 11 DEGs to the ‘pyruvate metabolism’, and 10 DEGs to the ‘terpenoid backbone biosynthesis’. For the susceptible rootstock ‘M9’ 126 DEGs belonged to the ‘metabolic pathway’ and 102 DEGs to ‘biosynthesis of secondary metabolites’. 22 DEGs in ‘M9’ were assigned to ‘phenylpropanoid biosynthesis’. Further 15 DEGs of ‘M9’ were associated to ‘biosynthesis of cofactors’, 11 to ‘biosynthesis of amino acids’ and 10 DEGs to ‘carbon metabolism’. 13 DEGs from ‘M9’ were assigned to ‘cystein and methionine metabolism’, 8 DEGs to ‘pyruvate metabolism’, and 8 DEGs to ‘terpenoid backbone biosynthesis’. Markedly lower numbers of DEGs for both genotypes were assigned to the remaining 85 pathways.

### Biotic Stress Response Is More Activated in the Susceptible Rootstock ‘M9’

Based on MapMan analysis, 44 DEGs were assigned to ‘biotic stress’. Most of these genes (26 DEGs) showed a higher fold change in ‘M9’ compared to Mr5. Four DEGs represent endochitinase EP3-like (*EP3,* MDP0000430546, MDP0000873235) and endochitinase-like (*HCHIB,* MDP0000710349, MDP0000224397) genes. Five copies of the pathogenesis-related thaumatin superfamily protein 1a (*AT1G20030,* MDP0000205389, MDP0000246775, MDP0000916930, MDP0000218699, MDP0000552328) showed a similar expression pattern. Downregulation in Mr5 and upregulation in ‘M9’ was found for six DEGs (MDP0000240781, MDP0000816743, MDP0000432882, MDP0000258248, MDP0000946278, and MDP0000746482) which were assigned to the disease resistance proteins of the TIR- nucleotide-binding site–leucine-rich repeat class (TIR-NBS-LRR) of plant resistance genes. The remaining DEGs, which were stronger upregulated in ‘M9’ compared to Mr5, were associated to different functions in the response to biotic stress (Reim et al., 2022, Dataset 2).

### Several Cell Wall Genes Are Differentially Expressed Between ‘M9’ and Mr5

Twenty-two DEGs were associated with cell wall biosynthesis. All of them were upregulated in ‘M9’ whereas nine DEGs were downregulated in Mr5. Out of these nine DEGs, two (MDP0000568045 and MDP0000126245) are homolog to the genes expansin-like A1 (*EXLA1*) and expansin-like A2 (*EXLA2*), whereas two other DEGs (MDP0000163930 and MDP0000218292) are homolog to genes encoding for pectin acetylesterase 8 (*PAE8*) that modifies cell wall pectin structure. Two further DEGs (MDP0000140678 and MDP0000398765) were associated to the xyloglucan endotransglucosylase/hydrolase genes *XTH16* and *XTH5* that are involved in cell wall remodeling.

### Hormone-Related Pathways and Signaling

#### Gibberellin Biosynthesis

Seventy-two DEGs were assigned to ‘hormone metabolism’. Eighteen of these DEGs were downregulated in ‘M9’, but upregulated in Mr5. Four genes are linked to the 2-oxoglutarate-dependent dioxygenase superfamily (2OGD), three genes (MDP0000810280, MDP0000210302, MDP0000120432) to *AOP3* and one to *AOP1* (MDP0000250956). 2OGDs are known to be involved in the biosynthesis of gibberellins (GAs). Similar expression patterns were obtained for six genes also associated with gibberellin biosynthesis: Three genes belong to gibberellin 20-oxidase (*GA20ox,* MDP0000280240, and MDP0000248981) and gibberellin 3-oxidase (*GA3ox,* MDP0000130015). Likewise, three genes were identified as gibberellin receptor *GID1b-like* (MDP0000929994 and MDP0000319522) and *GID1c-like* (MDP0000445131).

#### Signaling

Further DEGs that were upregulated in ‘M9’ and Mr5 belong to receptor-like kinases (RLKs) that are responsible for signaling and control a wide range of processes, *inter alia* disease resistance. Remarkably higher upregulation in Mr5 in this group was obtained for one gene (MDP0000172516) which was identified as G-type lectin S-receptor-like serine/threonine-protein kinase gene (*GsSRK*). Similar patterns were obtained for a PR5-like receptor kinase gene (*PR5*, MDP0000211661) and the putative receptor protein *PK1* (MDP0000270616). Ten DEGs that belong to RLKs were downregulated in ‘M9’ but not in Mr5. The gene MDP0000266451 encodes an L-type lectin-domain containing receptor kinase IX.1-like gene (*LecRK91*). Two further genes MDP0000144734 and MDP0000613625 are linked to the LRR receptor-like serine/threonine-protein kinase (*GSO1*). Upregulation in Mr5 but downregulation in ‘M9’ was also observed for a gene that encodes the wall-associated receptor kinase-like 20 protein (*WAKL20,* MDP0000676720).

The remaining DEGs related to hormonal pathways and signaling, are part of the abscisic acid (ABA), ethylene or cytokinin pathways. However, no obvious trend in gene expression pattern suggesting an involvement in plant response to ARD was discernible (Reim et al., 2022, Dataset 2).

### Secondary Metabolism

**Figure 4:**
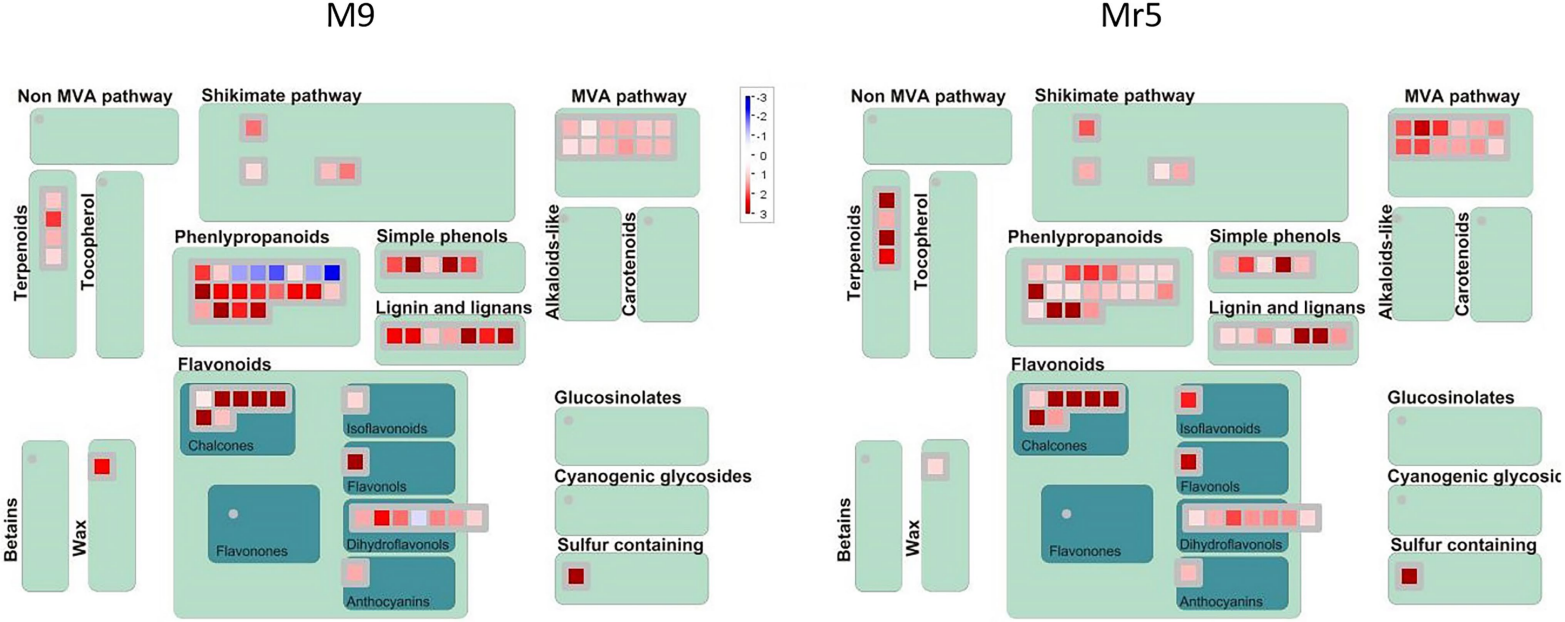
MapMan secondary metabolism overview of genes differentially expressed in ‘M9’ and *M.* ×*robusta* 5 (Mr5). The genes were assigned to 13 categories based on their expression pattern. A log_2_FC > 1 with a significance level of *p* < 0.05 indicates an upregulation (red), a log_2_FC < 1 with a significance level of *p* < 0.05 indicates a downregulation (blue). The intensity of the color represents the level of gene expression in the ARD soil relative to the γARD soil.

Sixty DEGs were associated to the secondary metabolites pathway, i.e., to chalcones, isoprenoids (mevalonate and terpenoids), phenylpropanoids and simple phenols (Figure 4).

A remarkable upregulation was observed for five DEGs (MDP0000257119, MDP0000208899, MDP0000432621, MDP0000287919, MDP0000302905) encoding for biphenyl synthase genes (*BIS*). The differences in regulation of these DEGs were rather low in between genotypes. However, a closer look at the normalized gene expression clearly indicated much higher expression levels of two ARD indicator genes in ‘M9’ than in Mr5 (Supplementary Figure 1).

Five DEGs of the phenylpropanoid synthesis pathway showed striking differences in expression pattern, as these genes were downregulated in ‘M9’ and upregulated in Mr5. These genes encode for HXXXD-type acyl-transferase family proteins. They were annotated as vinorine synthase-like gene (*ACT,* MDP0000269595), protein eceriferum 26 gene (*CER26-like,* MDP0000253113, MDP0000234868), and shikimate O-hydroxycinnamoyltransferase genes (*HST,* MDP0000197427, MDP0000177449).

Nine DEGs, which were attributed to the mevalonate pathway (MVA) were upregulated higher in Mr5 than in ‘M9’. They showed high sequence identity to the acetyl-CoA acetyltransferase *ACAT2* (MDP0000235454) and the hydroxymethylglutaryl-CoA synthase *HMGS* (MDP0000661951 and MDP0000138071). Two gene copies were annotated as mevalonate 5-diphosphate decarboxylase 2 genes (*MVD2,* MDP0000713057, and MDP0000135982). Three further DEGs (MDP0000894066,) were annotated as terpene synthase genes *TPS03* (MDP0000894066) and *TPS24* (MDP0000297049, MDP0000400627).

### Genes Related to Biotic Stress

Mapman analysis assigned 146 genes to the BIN group ‘Miscellaneous’. In this functional group several genes assumed to play a role in plant defense mechanisms against biotic stress showed different expression pattern in ‘M9’ and Mr5.

Four DEGs with strikingly higher expression in Mr5 were functionally assigned to phytocyanins (PCs) and identified as early nodulin like (*ENODL14,* MDP0000318341) and stellacyanin genes (MDP0000261622, MDP0000305092, and MDP0000878764). *ENODL14* probably regulates the symbiotic interaction between plants and microbes (Denancé et al., 2014).

Higher upregulation in Mr5 was also obtained for three DEGs associated with the senescence-specific cysteine protease *SAG12* (MDP0000222689 and MDP0000158144) and with the vacuolar-processing enzyme-like gene *ALPHA-VPE* (MDP0000188488). A gene that may also regulate apoptotic-like processes following pathogen attack was found to be downregulated in ‘M9’ but not in Mr5 and associated with the BAG (Bcl-2 associated athanogene) family molecular chaperone regulator 2 (*BAG2*, MDP0000305732). The metacaspase-1 gene *MC1* (MDP0000536363) was identified as another gene that plays an essential role in programmed cell death (PCD). However, *MC1* was upregulated in ‘M9’ but downregulated in Mr5.

Genes associated to the CBL-interacting serine/threonine-protein kinase 6-like gene *CIPK6* (MDP0000613342) and the β-glucosidase gene *BGLU12* (MDP0000133222) were found to be downregulated in ‘M9’, but upregulated in Mr5. *CIPK6* and *BGLU12* are known to be involved in the activation of phytohormones and defense related compounds (Tripathi et al., 2009a; Yang et al., 2021). Similar expression patterns were found for genes associated with the lysM domain receptor-like kinase 4 gene *LYK4* (MDP0000921828) and the wall associated kinase 4 gene *WAK4* (MDP0000167101). In *Arabidopsis LYK4* and *WAK4* serve as receptors during pathogen exposure (Kohorn and Kohorn, 2012; Wan et al., 2012b).

Interestingly, several DEGs that were higher upregulated in Mr5 than in ‘M9’ belong to the ubiquitin-proteasome system (UPS), which is a central regulator of many key cellular and physiological processes, including responses to biotic and abiotic stressors (Serrano et al., 2018). Two genes (MDP0000819881 and MDP0000237591) show sequence identity to the BOI-related E3 ubiquitin-protein ligase 3 gene (*BRG3*), whereas the gene MDP0000145991 shows identity to the Kelch-like protein encoding gene *AT1G16250*. The genes MDP0000208090, MDP0000843303, and MDP0000184157 seem to encode for F-box proteins (*FBP*), which mediate ubiquitination of proteins. Two further genes of the ubiquitin-proteasome system were downregulated in Mr5 but upregulated in ‘M9’; the E3 ubiquitin-protein ligase MIEL1-like gene (*MIEL,* MDP0000242922) and the U-box domain-containing protein 15-like (*PUB15,* MDP0000472519).

**Figure 5:**
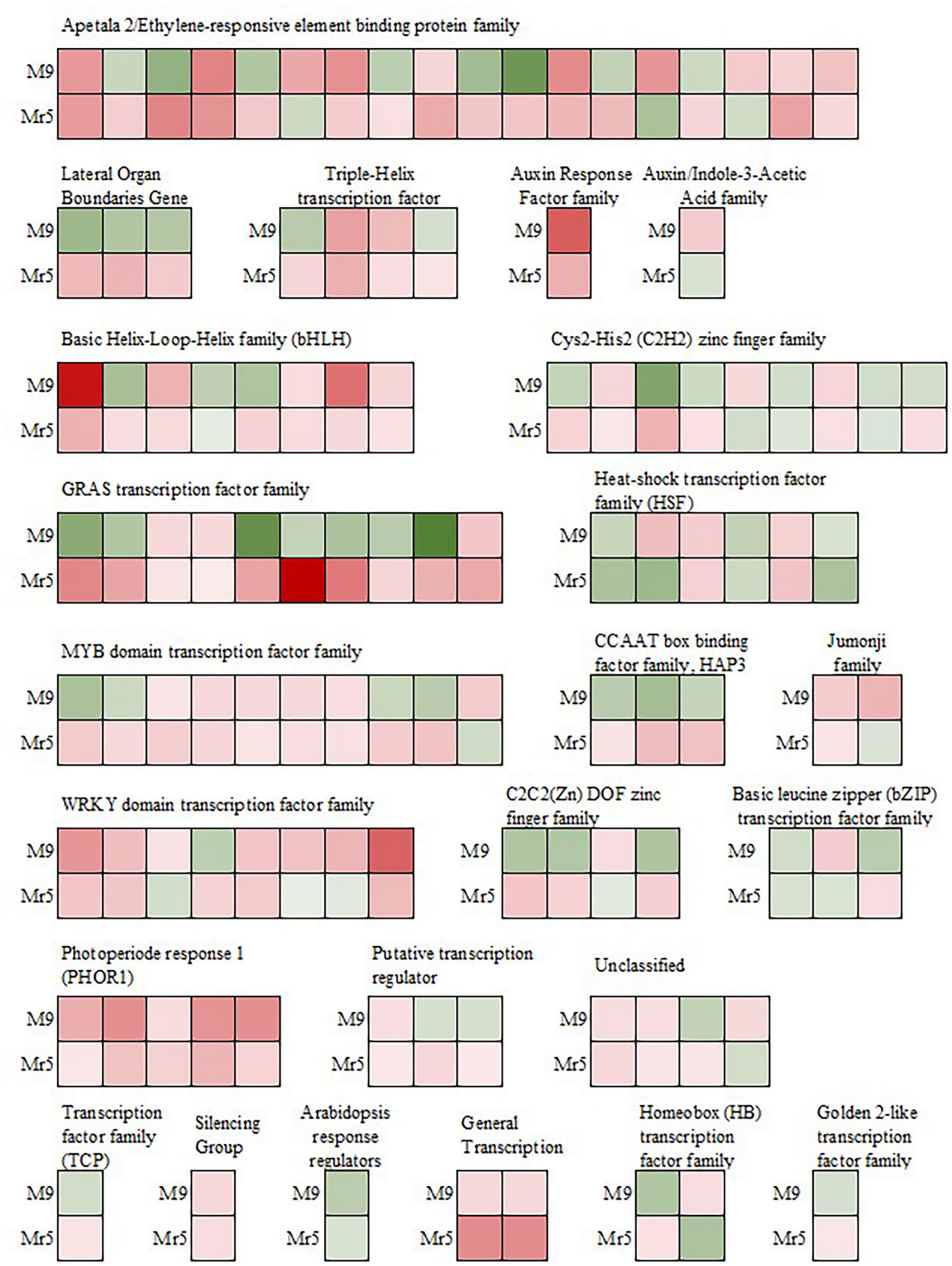
Expression of genes encoding transcription factors after induction of ARD in the susceptible rootstock ‘M9’ and the ARD tolerant wild apple cultivar *M.* ×*robusta* 5 (Mr5) within the 1,206 DEGs. Green indicates downregulation, whereas red indicates upregulation in ARD soil. The intensity of the color represents the level of gene expression. *GRAS:* named after the first three transcription factors, namely, GAI (Gibberellic Acid Insensitive), RGA (Repressor of Gai) and SCR (Scarecrow); *MYB*: Avian myeloblastosis virus; *CCAAT box*: distinct pattern of nucleotides with GGCCAATCT consensus sequence; *HAP3*: Heme Activator Protein3; *WRKY*: named after the WRKYGQK heptapeptide at the N-terminal end; *C2C2(Zn) DOF*: family of DNA binding with one C2C2-type zinc finger motif at the N-terminus.

Many transcription factors (TF) are known to play important roles in regulating plant resistance mechanisms. In the present study, 110 DEGs belonging to 24 different transcription factor families were identified (Figure 5). In ‘M9’ about half of these TFs (55) were upregulated, while in Mr5, the majority of TFs (87) were upregulated.

Most of the downregulated TFs in ‘M9’ belong to the GRAS transcription factor family and are associated with DELLA proteins (*RGL2*, MDP0000245253 and MDP0000522931; *RGL1*, MDP0000768445; Reim et al., 2022, Dataset 2). In the *AP2/EREBP* transcription factor family, seven genes probably associated with stress response, were downregulated in ‘M9’, but upregulated in Mr5. Three genes (MDP0000128326, MDP0000177547, and MDP0000276536) are linked to the ethylene-responsive transcription factor *RAP2.11* and two genes (MDP0000517257, MDP0000246184) encode for the ethylene-responsive transcription factors *ERF02* and *ERF062*. Two copies of the ethylene-responsive transcription factor TINY-like (*TINY2,* MDP0000790788, MDP0000242611) were also upregulated in Mr5 but not in ‘M9’.

In the MYB-related transcription factor family four genes (MDP0000249611, MDP0000152575, MDP0000887107, and MDP0000931057) were also differently expressed since they were downregulated in ‘M9’, but upregulated in Mr5. These genes encode the transcription factors *MYB4*, *MYB18* (or *LAF1*), *MYB123* (or *TT2*), and *MYB5*. Three TFs (MDP0000818967, MDP0000821908, MDP0000936873) of the CCAAT box binding factor family (*CBF*) showed also a upregulation in Mr5 but not in ‘M9’. In contrast, TFs apparently involved in pathogen response (*WRKY* and *HSF*) were more strongly upregulated in ‘M9’ or equally strong in ‘M9’ and Mr5. One exception was MDP0000168871 encoding for the WRKY DNA-binding protein 14 (*WRKY14*).

### Detoxification Pathways

Several genes differently expressed between ‘M9’ and Mr5 seem to play a role in detoxification pathways. Four DEGs with higher upregulation in Mr5 were assigned to the Cytochrome P450 superfamily (*CYP*): *CYP714A1* (MDP0000124807), *CYP71A25* (MDP0000205620), *CYP98A3* (MDP0000533607) and *CYP93D1* (MDP0000214215). One gene that appeared to encode a glutathione S-transferase (*GSTU19,* MDP0000558468) was highly upregulated in both genotypes but with remarkably higher upregulation level in Mr5. Three further DEGs (MDP0000176250, MDP0000280885, and MDP0000318032) belonged to the UDP-glycosyltransferase family (UGTs). The first two genes were downregulated in ‘M9’, whereas no regulation was detected in Mr5; the latter gene was upregulated in Mr5 only, while ‘M9’ showed no gene regulation. Genes of this family are known to contribute to detoxification. Similar contribution to detoxification processes was assumed for MDP0000175055, a gene that was assigned to the protein detoxification 35-like (*DTX35*). Two genes possessed function in antioxidant processes and were connected to polyamine oxidase 5 (*PAO5,* MDP0000941459) and the tetrapyrrole-binding protein (*GUN4,* MDP0000906703). Both *DTX35* and *PAO5* as well as *GUN4* were downregulated in ‘M9’ and upregulated in Mr5.

### Protein–Protein Interaction

**Figure 6:**
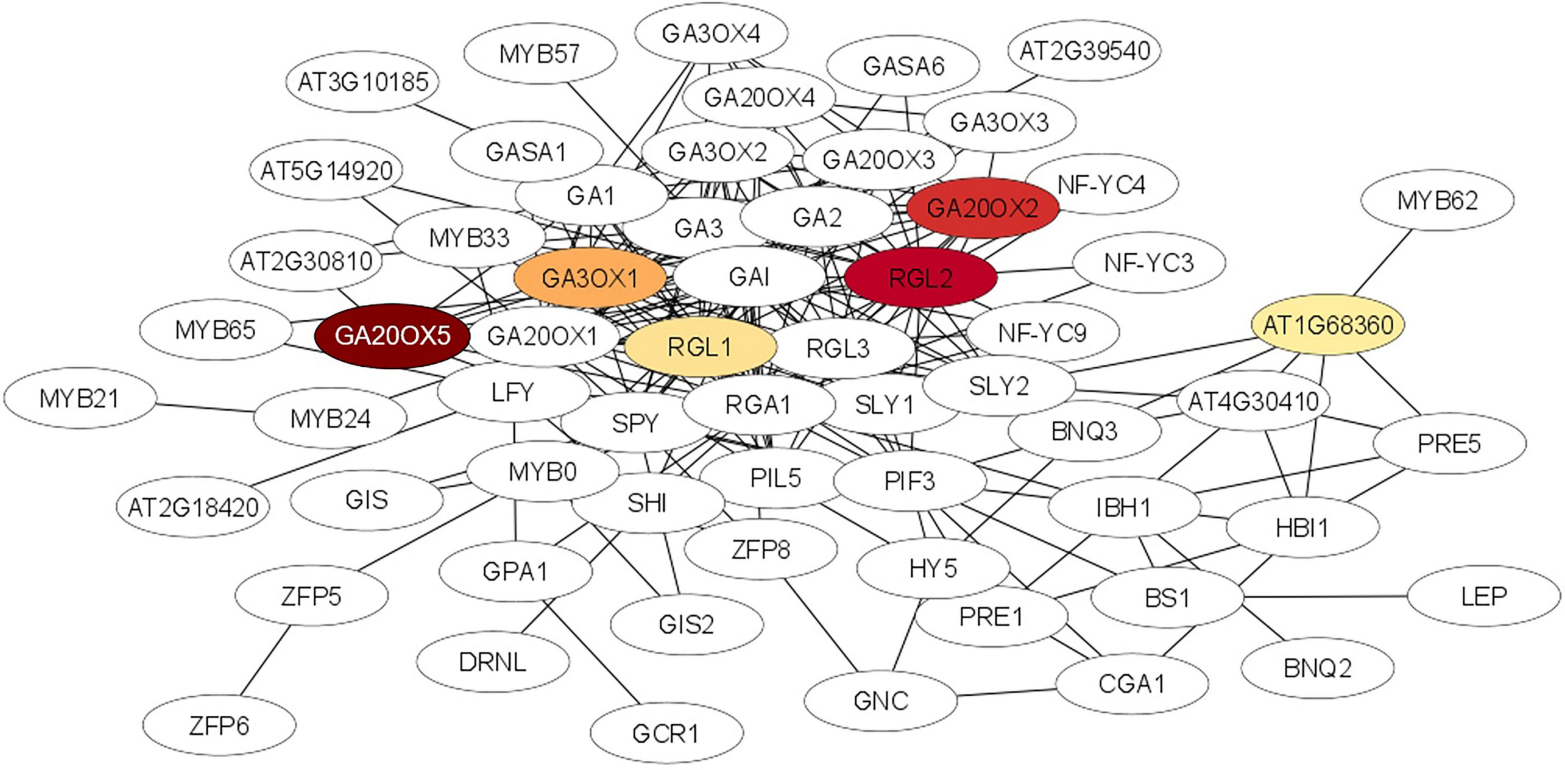
Protein–protein interaction network for the gibberellic acid mediated signalling pathway which was significantly enriched (FDR < 0.001) after STRING analysis using the STRING database (http://string-db.org/). Nodes represent proteins, lines represent interactions between proteins. The intensity of the red color of the node represents the degree of upregulation of the gene in ARD tolerant wild species Mr5 compared to the susceptible rootstock ‘M9’. Nodes without a color were not differentially expressed or showed no differences in fold change values between ‘M9’ and Mr5. The network was generated using the Cytoscape tool.

A STRING analysis was performed to investigate potential interactions between proteins encoded by the DEGs. Two gene ontology (GO) terms were significantly enriched (FDR < 0.001) in the STRING network ‘biological process’. The processes belong to the gibberellic acid mediated signaling pathway (GO:0009740; ES = 5.17) and the gibberellin mediated signaling pathway (GO:0010476, ES = 4.27). The list of the 50 highly interactive proteins of each group is given in Supplementary Table 2. The gibberellic acid mediated signaling network was visualized using the Cytoscape software. As shown in Figure 6 the proteins of six DEGs of our entire data set were connected with most proteins of the GA network and five of them (*GA20OX5*, *GA20OX2*, *RGL2*, *RGL1* and *GAOX1*) were in the center of the interaction network.

### RT-qPCR Validation

**Figure 7:**
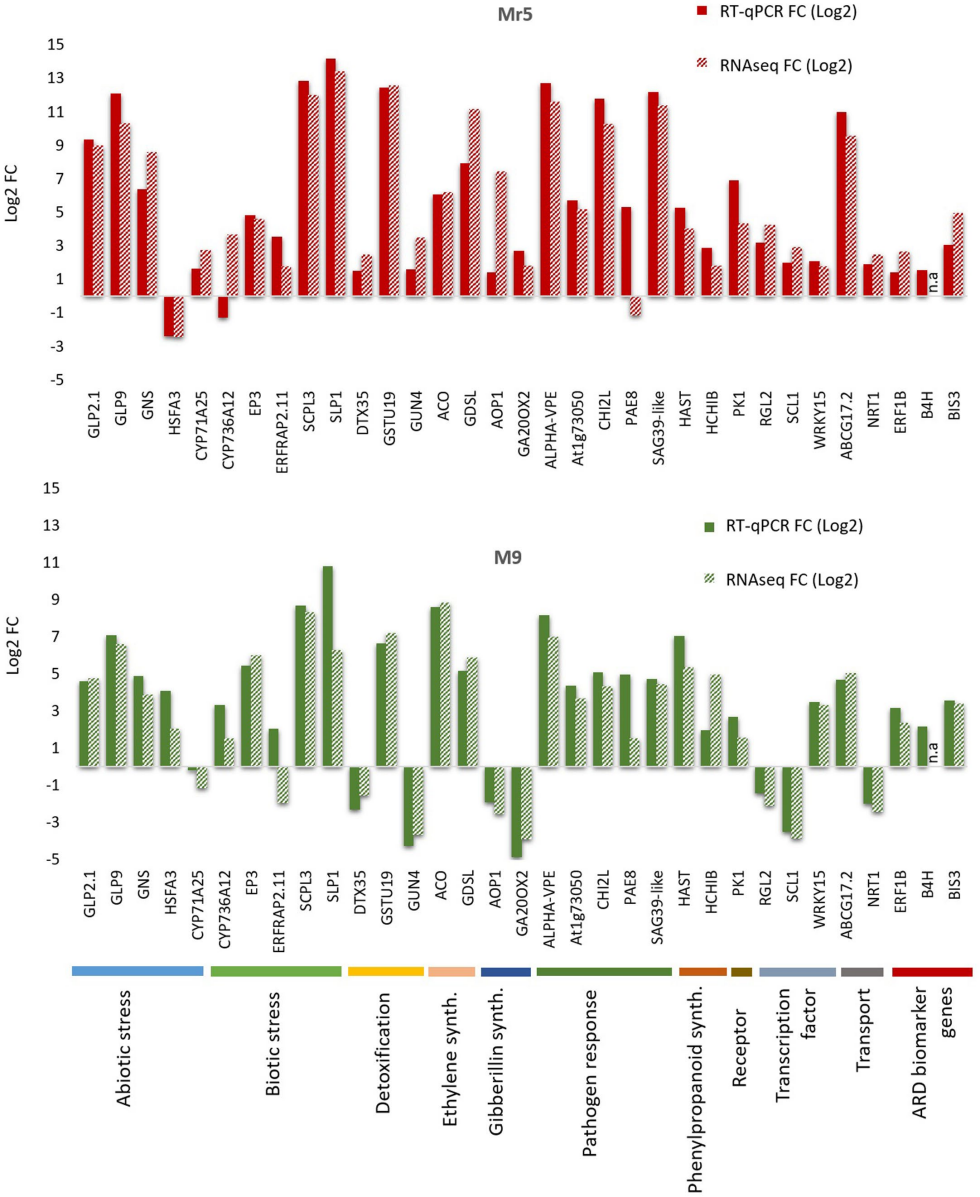
Illustration of the results of the RT-qPCR validation for the selected 30 differentially expressed genes and the three ARD biomarker genes biphenyl synthase 3 (*BIS3*), biphenyl 4-hydroxylase (*B4H*) and ethylene-responsive transcription factor 1B-like (*ERF1B*). The 30 DEGs belong to 10 different major functions, as illustrated by the different colors at the bottom of the figure. The 30 DEGs thought to play a potential role in the defense response to ARD were validated for the ARD tolerant apple cultivar Mr5 (red bars) and the susceptible rootstock ‘M9’ (green bars). A highly significant positive correlation (*r* = 0.91; *p* < 0.0001) between the log2 fold change of the RNA-seq and RT-qPCR results was observed. n.a: not analyzed.

To validate the RNA-seq results, 30 DEGs possibly associated with a response to ARD were selected for quantitative real-time PCR (RT-qPCR) along with 3 ARD biomarker genes (*B4H, BIS3* and *ERF1B* Rohr et al. 2020). All selected DEGs showed concordant expression patterns between RNA-seq and RT-qPCR results with two exceptions in Mr5 (*CYP736A12; PAE8*) and two exceptions in ‘M9’ (*ERFRAP2.11* and *CYP71A25*; Figure 7). Despite of those exceptions a highly significant positive correlation (*r* = 0.91; *p* < 0.0001) between the log_2_ fold change of the RNA-seq and RT-qPCR results was observed.

## Discussion

The knowledge of the molecular response of apple to ARD is still scarce. First transcriptomic studies have either focused on one apple rootstock genotype (Weiß and Winkelmann, 2017; Weiß et al., 2017a) or analyzed gene expression profiles after inoculations with single pathogens contributing to the ARD complex (Shin et al., 2016; Zhu et al., 2019; Xiang et al., 2021). Therefore, in this study a comparative RNA-seq approach was performed in order to identify tolerance or sensitivity traits at the molecular level that could be useful in breeding rootstocks that are less susceptible to ARD. It was decided to work with ARD-affected soils here, since it was assumed that the interaction of the various causal agents provokes a different and most likely stronger reaction. The plant growth data indicated a moderate ARD severity for the soil used in this study, resulting in a significantly reduced shoot fresh mass in the ARD sensitive genotype ‘M9’ (Figure 1). More than 1,000 statistically significant differentially expressed genes that belong to several functional groups have been identified. Numerous of these DEGs appeared to play a role in plant defense against biotic stress.

### ‘M9’ and Mr5 Differ in Their Activation of Pathogen Response Genes

Surprisingly, most DEGs in the functional group ‘biotic stress’ were more induced in ‘M9’ than in Mr5. Two DEGs within this functional group coded for the chitinases *EP3* and *HCHIB*. Chitinases were often considered pathogenesis-related (PR) proteins (Passarinho and de Vries, 2002) and chitinase genes *CHIA* (Weiß and Winkelmann, 2017) and *CHIB* (Reim et al., 2020) have already been shown to be upregulated in roots of the rootstocks ‘M26’ and ‘M9’ in response to ARD. However, upregulation of chitinases do not always result in an increased resistance to pathogens. Therefore, it was assumed that some chitinase genes could play a role in other plant functions such as growth or developmental (Passarinho and de Vries, 2002). Particularly *EP3* was described as involved in the biogenesis of the cell wall and its expression was found in root epidermis and emerging root hairs (Passarinho et al., 2001; Vanholme et al., 2014). The *HCHIB* has multiple functions and could play a role in plant defense but may also be involved in cell wall macromolecule catabolic processes (Vanholme et al., 2014). Since the higher expression of *EP3* and *HCHIB* in ‘M9’ was contrary to the ARD susceptible phenotype of this rootstock, it was assumed that these genes were not involved in a direct defense reaction to ARD or they were not effective against the causative pathogens. Similar conclusions can be made for the TIR-NBS-LRR genes (*TNL*s) and the thaumatin-like protein genes (*TLP*s) that were also expressed more strongly in ‘M9’ than in Mr5. In other studies on apple rootstocks ‘M26’ and ‘M9’ it was shown that the thaumatin-like protein gene *TL1* was significantly upregulated in the roots in response to ARD (Weiß et al., 2017a; Reim et al., 2020). Despite clear evidence of their function in plant defense response (reviewed in Liu et al., 2010), no relationship between an increased ARD tolerance and *TLP* and *TNL* expression, respectively was found in the present study. These results suggest that other genes, in addition to the biotic stress genes mentioned above, may lead to increased ARD tolerance in Mr5.

Numerous other genes potentially involved in pathogen response were more upregulated in Mr5 than in ‘M9’ after ARD induction. These included *SAG12*, *ALPHA-VPE* and *BAG2,* which are involved in programmed cell death (PCD; Doukhanina et al., 2006; Zhang et al., 2010; Singh et al., 2013). PCD includes several types of plant reactions such as hypersensitive response (HR), apoptotic-like processes and senescence to operate against biotic stress (Reape and McCabe, 2008). *SAG12* is a senescence marker that is induced during hypersensitive response (HR). HR, in turn, induces the expression of *VPE* (Pontier et al., 1999), which functions as the execution organ of cell death. *BAG2* also likely regulates apoptosis-like processes following pathogen attack (Doukhanina et al., 2006). These results suggested that PCD may lead to increased ARD tolerance, as tightly controlled PCD can promote plant survival by restricting pathogen growth (Reape and McCabe, 2008; Burke et al., 2020).

Several genes of the ubiquitin-proteasome system (*UPS*) were upregulated more strongly in Mr5 than in ‘M9’. *UPS* is an important system that maintains cellular events by purging misfolded or damaged proteins; which is also a central regulator of plant immunity (Abd Hamid et al., 2020). Kelch-like (*KLHL*s) and F-box proteins (*FBP*) are responsible for the specific ubiquitination of undesired proteins and therefore directly involved in plant defense (Abd Hamid et al., 2020). The BOI-related E3 ubiquitin-protein ligase 3 (*BRG3*) gene represents a subclass of RING E3 ligases and has a common role in the control of cell death (Luo et al., 2010). A study in *Arabidopsis* clearly demonstrated that *BOI-RELATED GENES* (*BRG*s) contribute to *Botrytis cinerea* resistance and suppression of pathogen- or stress-induced cell death (Luo et al., 2010). In contrast, the two UPS genes *MIEL1* and *PUB15* were downregulated in Mr5 but upregulated in ‘M9’. *MIEL1* acts as a negative regulator of plant resistance and many U-box E3 ligase genes were also downregulated by fungal infection (Duplan and Rivas, 2014). Further investigation of additional UPS genes are necessary to reveal a possible relationship with ARD tolerance.

Downregulation in ‘M9’ and upregulation in Mr5 was found for several genes that are possibly involved in immune signalling (*CIPK6, LYK4, WAK4*; Wan et al., 2012a; Sardar et al., 2017; Kohorn et al., 2021). Calcineurin B-like proteins (CBLs) interact with CBL-interacting protein kinases (CIPKs) to form a CBL–CIPK signaling network. This signalling pathway represents a central system of hormone signaling to mediate plant responses to a variety of external stresses and there is increasing evidence for a role in immune signaling (Ma et al., 2020). Studies on the role of *CIPK*6 in *Arabidopsis* showed that *CIPK6* is probably involved in root growth and functions as an auxin transporter (Tripathi et al., 2009a,b). *LYK4* belongs to the lysin motif receptor-like kinases (RLKs) and plays a role in the chitin recognition receptor complex to assist in defense responses against pathogenic fungi (Wan et al., 2012a; Buendia et al., 2018; Xue et al., 2019). Thus, higher expression of *LYK4* in Mr5 may promote defense against fungi suspected to be involved in ARD, such as *Nectriaceae* (Grunewaldt-Stöcker et al., 2019; Popp et al., 2019, 2020; Grunewaldt-Stocker et al., 2021). The *Arabidopsis* cell wall–associated RLK gene *WAK4* is part of the wall associated kinase (WAK) gene family and may also function as a signalling receptor during pathogen exposure as shown for the bacterium *Pseudomonas syringae* in tomato (Zhang et al., 2020). It is also conceivable that the upregulation of *WAK4* promotes defense against bacteria that were part of the ARD complex such as, e.g., *Streptomyces* spp. (Mahnkopp-Dirks et al., 2021). However, *WAK4* may also only serve a role in cell elongation and be required for plant development (Kohorn and Kohorn, 2012). Further studies on RLK genes are needed to reveal a possible role in plant-microbe interactions in defense responses to ARD.

Four phytocyanins (PC) associated genes were conspicuously expressed more highly in Mr5, suggesting their involvement in ARD response. PCs are ancient blue copper-binding proteins in plants that function as electron transporters and play an important role in plant development and stress resistance (Ma et al., 2011). PCs are divided in subclasses that include *inter alia* early nodulin-like proteins (*ENODL*) or stellacyanins (SCs; Ma et al., 2011). It is known that *ENODL* gene expression is induced during the establishment of symbiosis between *Rhizobium* and plants (Denance et al., 2014). Although nitrogen-fixing nodulation is normally found only in legumes, several non-nodulating plant species also contain noduline-like proteins (Denance et al., 2014). In *Arabidopsis* it was demonstrated that early nodulin-like activities play an important role in plant immunity (Denance et al., 2014). In contrast, the exact function of stellacyanins is still unrevealed. Future studies are necessary to elucidate the involvement of nodulin-like proteins in plant–microbe interactions and a possible function in ARD response.

### Metabolism of Volatile and Nonvolatile Compounds Is Increased in Mr5

Different genes involved in secondary metabolism were selectively regulated in their expression when comparing apple plantlets grown in ARD soil and those grown in γARD soil. Most of the DEGs were categorized to the phenylpropanoid and mevalonate (MVA) pathway. MVA is responsible for terpenoid biosynthesis (Wang et al., 2019). Terpenoids represent a large and diverse group of volatile and nonvolatile compounds (Abbas and Yu, 2017).

Several volatiles are emitted by plants and are important chemical signals for the activation of plant defense against biotic and abiotic stresses (Singh and Sharma, 2015; Balan et al., 2018). It is well documented that volatiles are also emitted by the roots in the plant rhizosphere and play important ecological roles in the soil ecosystem (Delory et al., 2016). For example, root-produced volatiles can act as foraging signals for entomopathogenic nematodes that parasitize insects and may function as biological insecticides (Turlings et al., 2012). Studies on nematode communities in ARD soils showed that the free living nematode genera *Acrobeloides* and *Cephalenchus* were significantly increased in abundance in ARD soils, whereas *Steinernema* an entomopathogenic nematode was largely overrepresented in control plots (Kanfra et al., 2018, 2022a,b). Most of the volatile terpenoids were found accumulated in the roots and play diverse roles in beneficial interactions and in mediating antagonists among organisms such as entomopathogenic nematodes (Abbas and Yu, 2017). For example, Caryophyllene is known to be an herbivore-induced belowground signal, which strongly attracts entomopathogenic nematodes (Rasmann et al., 2005; Delory et al., 2016). Possibly, a higher expression of terpenoid volatiles in Mr5 may attract beneficial soil organisms, such as entomopathogenic nematodes, and thereby has an effect on ARD severity. However, further studies are needed to clarify a possible role of terpenoid volatiles in ARD.

### Nonvolatile Terpenoids Are Also Involved in Different Defense Responses

Several studies reported that nonvolatile terpenoids can be exuded from roots into the rhizosphere and the surrounding soil environment where they *inter alia* act as phytoalexines (reviewed in Tholl, 2015). Furthermore, a study on the functional potential of the microbiome in ARD soils showed that genes of mevalonate-based synthesis of terpenoids were upregulated in microorganisms of the ARD rhizosphere (Radl et al., 2019). These microorganism-produced regulatory enzymes of MVA metabolism can transduce endosymbiotic microbial signals into the host and thus play a key role in symbiotic signal transduction between the host plant and rhizobia and arbuscular mycorrhiza fungi (Venkateshwaran et al., 2015). However, a relationship between ARD severity and the production of terpenoid phytoalexins in the plant has not been observed to date. Several studies on ARD demonstrated remarkably higher contents of biphenyl and dibenzofuran phytoalexins in plants cultivated in ARD soils compared to disinfected ARD soils (Winkelmann et al., 2019; Reim et al., 2020; Rohr et al., 2020b; Balbin-Suarez et al., 2021; Busnena et al., 2021). However, Reim et al. (2020) showed that the genes coding for biosynthesis of these phytoalexins were more strongly induced in the susceptible rootstock ‘M9’ than in the ARD tolerant genotype Mr5, indicating that these phytoalexin compounds do not help against ARD. One possible explanation could be that the produced phytoalexins are not only harmful to the pathogen but also to the plant if they cannot be detoxified by the plant after the threat has been eliminated (Camagna et al., 2020).

### Gibberellin Coordinated Plant Immune Response and Detoxification Processes Are Increased in Mr5

Several genes involved in gibberellin biosynthesis were differentially expressed in apple plantlets grown in ARD soil when compared to γARD soil and the gibberellic acid mediated signaling pathway was demonstrated to be enriched in our study. Furthermore, a large part of the genes was differently regulated between Mr5 and ‘M9’ indicating a genotype-specific response. Gibberellins (GAs) and DELLAs, the key negative regulators of GA, have been intensively studied in the context of plant growth and development. However, different studies indicated that the gibberellic acid mediated signaling played ambiguous roles in the plant innate immune signaling network (reviewed in De Bruyne et al., 2014). In our study, the majority of genes involved in the gibberellic acid mediated signaling pathway were upregulated in the ARD tolerant genotype Mr5 but not in the susceptible rootstock ‘M9’. This was particular true for genes encoding DELLA proteins, the gibberellin receptor *GID1b-like* and genes encoding gibberellin oxidase genes (*GA2OX6, GA20OX2, GA20OX1, GA3OX1*). Investigations on GA-mediated immunity have shown that DELLAs modulate reactive oxygen species (ROS) production (Achard et al., 2008). Upon pathogen attack, ROS production can dramatically increase to exert antimicrobial actions against a broad range of pathogens (Fang, 2011). On the other hand, high levels of ROS may result in significant damage to cell structures (Matés, 2000). Whereas GA can induce ROS (Ishibashi et al., 2012), the gibberellin receptor *GID1b-like* and DELLAs (e.g., *RGL1* and *RGL2*) elevate the induction of a subset of antioxidant genes involved in ROS detoxification such as glutaredoxin genes and glutathione *S*-transferase (e.g., *GSTU19*), which were remarkably upregulated in this study (Xia et al., 2015; Ozyigit et al., 2016; Sharma et al., 2021). Thereby, ROS levels were reduced and plant cell death was restrained (Achard et al., 2008; Hauvermale et al., 2014). GA oxidases (GAoxs) also play key roles in the deactivation of bioactive GA levels (Huang et al., 2015). Thereby, GAoxs and DELLAs contribute to an enhanced disease resistance by fine-tuning ROS (De Bruyne et al., 2014). The findings of the present study led us to speculate that some genes of the gibberellin biosynthesis and signaling pathways might play a role in the increased tolerance of Mr5 to ARD.

Additional genes involved in ROS detoxification processes were also upregulated after growth in ARD soil, particularly in Mr5 (*CYP450, GSTs, UGTs*, *DTX35, PAO5, GUN4*, and *BGLU12*). The majority are described as being involved in the protection of the plant against oxidative stress damage by ROS. For example the CYP450 genes *CYP714A* and *CYP71A25,* which were all more induced in Mr5 in this study, are assumed to be involved in deactivation of bioactive components (Xu et al., 2015; Hao et al., 2018; Khanom et al., 2019; Pandian et al., 2020). Furthermore, several studies recognized that *GST* plays an important role in the detoxification and maybe also a crucial role in plant antioxidative defense by limiting ROS after pathogen attack (Gullner et al., 2018). *DTX35* is another gene that is reported to minimize the effects of oxidative stress by regulating the amount of ROS (Lu et al., 2019). The same was demonstrated for *PAOs* (Andronis et al., 2014), *GUNs* (Larkin, 2016) and *BGLU12* (Baba et al., 2017). In contrast, UDP-glycosyltransferase family genes (*UGT*s) are reported to contribute to the detoxification of mycotoxins (Poppenberger et al., 2003). Studies in wheat clearly demonstrated that the resistance to *Fusarium* was enhanced by upregulation of *UGT*s (Poppenberger et al., 2003; He et al., 2018). Eventually, the higher expression of genes responsible for detoxification processes of the plant’s own and endogenic toxic compounds, for instance the biphenyls and dibenzofurans mentioned above, in Mr5 lead to higher ARD tolerance in this genotype.

### ARD Induced Cell Wall Stabilization in Mr5

GA-induced metabolic changes may also result in cell wall modification. The plant cell wall plays an important role in plant immunity since it is the first physical barrier that plant pathogens must overcome. Fungi, bacteria and nematodes need to degrade the plant cell wall during their infection process to obtain nutrients for their growth. Plants have developed a pathogen defense system with which they activate cell wall remodeling to sustain the cell wall integrity to prevent disease (Bellincampi et al., 2014; Bacete et al., 2018). GA and DELLA have long been shown to, respectively, induce and repress cell wall relaxation by altering the expression of xyloglucan endotransglucosylase/endohydrolases (XTHs) and expansins, so called cell wall degrading enzymes (CWDEs; Miedes and Lorences, 2007; Park et al., 2013; Park and Cosgrove, 2015). Interestingly, a large proportion of these genes that function as CWDEs were downregulated in Mr5 but not in ‘M9’. Two of them belong to expansins (*EXLA1; EXLA2*) and two were identified as xyloglucan endotransglucosylase/hydrolase genes *XTH16* and *XTH5*. Expression of *XTH* loosens the cell wall by hydrolyzing cell wall components (Rose et al., 2002), whereas expansin does it by disrupting hydrogen bonding (Cosgrove, 2000; Ogawa et al., 2003). Although cell wall loosening is essential during growth and development, it may render the plant more vulnerable to pathogen entry or allowing enhanced nutrient leakage (De Bruyne et al., 2014). Suppression of CWDEs prevents the cell wall from loosening and maintains the stability of the cell wall. The pectin acetylesterase 8 (*PAE8*) is also involved in cell wall functions and was downregulated in Mr5 but not in ‘M9’. Pectin acetylesterases (PAEs) are involved in enzymatic deacetylation of pectin and are key determinants for functional integrity of plant cell walls (Philippe et al., 2017; Gigli-Bisceglia et al., 2020). Independent studies reported that downregulation of PAEs increased the stiffness of cell walls and impaired the digestibility of pectin to muster effective immune responses against pathogens (Gou et al., 2012; Orfila et al., 2012; Gigli-Bisceglia et al., 2020; Li et al., 2020). Based on these observations we speculated that the downregulation of CWDEs in Mr5 improved the stabilization of root cell walls, which protected this genotype against soil borne pathogen attack and consequently increase the ARD tolerance.

### Genes Involved in Lignin Biosynthesis Are Upregulated in Mr5

Numerous DEGs in this study belonged to the phenylpropanoid pathway, which was already described as being induced by fungal pathogens in *Malus* (Balan et al., 2018). Furthermore, several MYB transcription factors were observed to be differentially expressed in this study. MYB TFs are known to regulate the synthesis of phenylpropanoid-derived compounds (Liu et al., 2015). After pathogen attack of the plant cell wall, the phenylpropanoid defense pathway was triggered, which in turn provided the lignin-building monolignols. The production of lignin has significant effects on physicochemical properties of the cell wall, because lignin protects the cell wall polysaccharides from microbial degradation (Liu et al., 2015). Thus, its biosynthesis can defend the plant against various biotic and abiotic stresses, such as wounding, pathogen infection, metabolic stress, and perturbations in cell wall structure (Liu et al., 2015).

## Conclusion

Comparative transcriptome analysis of apple plants cultivated in ARD soil and disinfected ARD soil allowed the identification of numerous DEGs associated with plant stress response and potentially involved in a response to ARD. The comparison of gene expression between the susceptible rootstock ‘M9’ and the ARD tolerant wild species Mr5 gave indications which of these differentially expressed genes are possibly more effective to combat ARD.

In this study, 1,206 genes were found to be differentially expressed in response to ARD. At the same time remarkable differences in gene expression between both genotypes were observed for a number of genes indicating a genotype-specific response to ARD. That was especially true for genes that were categorized to the GA biosynthesis and signaling, which were significantly enriched. Most genes connected to GA biosynthesis were associated with functions in cell wall stabilization. These genes and further single genes also involved in cell wall lignification were remarkably downregulated in Mr5 suppressing cell wall relaxation and maintaining a stable cell wall. This leads to the assumption that cell wall stabilization is one of the possible strategies to reduce the negative effects of ARD on the plant. Furthermore, several genes connected to GA mediated detoxification of ROS and genes generally associated to detoxification processes were also more induced in Mr5. These findings likewise let us assume that the support of detoxification processes contribute to a reduced susceptibility to ARD. Furthermore, genes which are expressed higher in Mr5 were associated with the metabolism of volatile and nonvolatile compounds and several pathogen-related genes. Overall, the results of this study suggest that the ARD tolerance in Mr5 is not based on one single mechanism, but rather requires the activation of numerous defense mechanisms. However, since the composition of the ARD pathogen complex and the dysbiotic state of different ARD soils differ, further investigations must include more ARD soils and also earlier time points.

## Data Availability Statement

The datasets presented in this study can be found in online repositories (Reim et al., 2022). The names of the repository/repositories and accession number(s) can be found in the article/Supplementary Material.

## Author Contributions

SR, AC, and ADR analyzed the data. SR wrote the manuscript. TW, SR, ADR, and HF were involved in performing the experiments. TW and HF assisted in drafting the manuscript and conceived and coordinated the project. All authors contributed to the article and approved the submitted version.

## Funding

This work was part of the project BonaRes-ORDIAmur funded by the German Federal Ministry of Research and Education within the frame of the program BonaRes (grant nos. 031B0025A and 031B0025B).

## Conflict of Interest

The authors declare that the research was conducted in the absence of any commercial or financial relationships that could be construed as a potential conflict of interest.

## Publisher’s Note

All claims expressed in this article are solely those of the authors and do not necessarily represent those of their affiliated organizations, or those of the publisher, the editors and the reviewers. Any product that may be evaluated in this article, or claim that may be made by its manufacturer, is not guaranteed or endorsed by the publisher.

## Footnotes

^1^ http://www.usadellab.org/cms/?page=trimmomatic

^2^ https://mapman.gabipd.org/download

^3^ https://www.ncbi.nlm.nih.gov/sites/batchentrez

^4^ https://www.kegg.jp/kegg/tool/map_pathway1.html

^5^ http://string-db.org/

## Supplementary Material

Supplementary Table 1 KEGG input and output data for susceptible rootstock ‘M9’ and the ARD tolerant wild apple accession *M. ×robusta* 5 (Mr5).

Supplementary Table 2 String input and output for the susceptible rootstock ‘M9’ and the ARD tolerant wild apple accession *M. ×robusta* 5 (Mr5).

Supplementary Table 3 Results of the primer validation.

Supplementary Figure 1 Normalized gene expression of the three biomarker genes biphenyl synthase 3 (*BIS3*), biphenyl 4-hydroxylase (*B4H*) and ethylene-responsive transcription factor 1B-like (*ERF1B*) for the susceptible rootstock ‘M9’ and the ARD tolerant wild apple accession *M. ×robusta* 5 (Mr5) after cultivation in ARD soil and γARD soil, respectively.
