# Supplementary figures and images for: Identification of Candidate Genes Associated With Tolerance to Apple Replant Disease by Genome-Wide Transcriptome Analysis
Source: Front Microbiol. 2022 May 9;13:888908. doi: 10.3389/fmicb.2022.888908 (PMC9125221; doi:10.3389/fmicb.2022.888908)

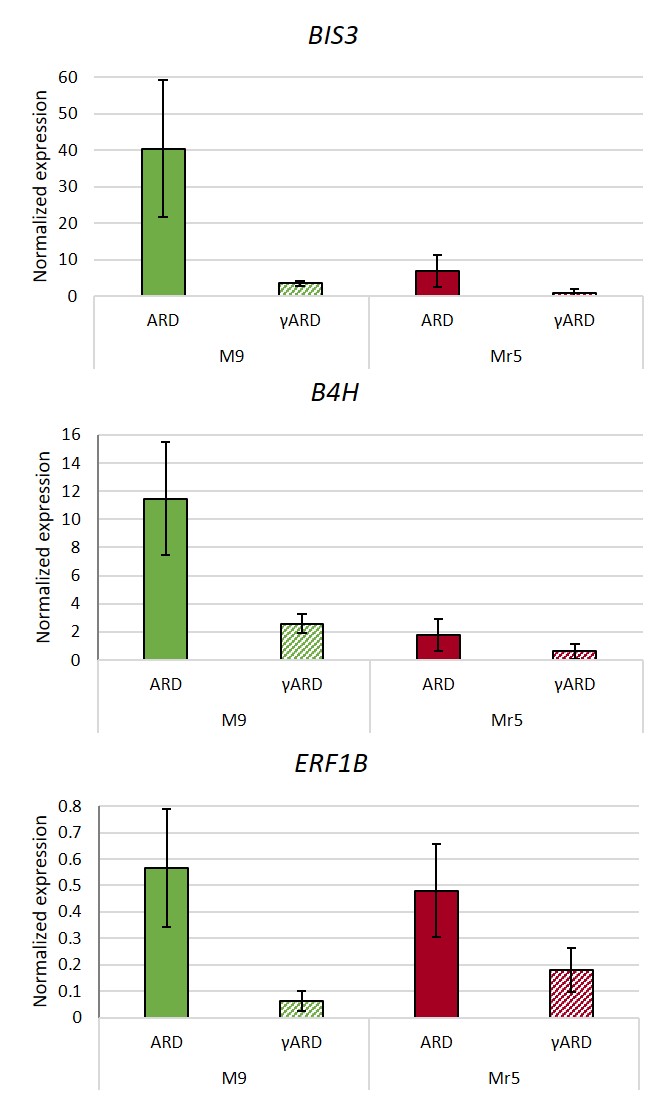

Supplement: Supplementary Figure 1 — Normalized gene expression of the three biomarker genes biphenyl synthase 3 (BIS3), biphenyl 4-hydroxylase (B4H) and ethylene-responsive transcription factor 1B-like (ERF1B) for the susceptible rootstock ‘M9’ and the ARD tolerant wild apple accession M. ×robusta 5 (Mr5) after cultivation in ARD soil and γARD soil, respectively. [file Image_1.JPEG]
